# Screening for periodontal diseases by non-dental health professionals: a protocol for a systematic review and overview of reviews

**DOI:** 10.1186/s13643-019-0977-9

**Published:** 2019-02-25

**Authors:** Birgit Teufer, Isolde Sommer, Barbara Nussbaumer-Streit, Viktoria Titscher, Corinna Bruckmann, Irma Klerings, Gerald Gartlehner

**Affiliations:** 1 Department for Evidence-based Medicine and Clinical Epidemiology, University for Continuing Education Krems (Danube University Krems), Dr. Karl Dorrek Str. 30, 3500 Krems, Austria; 2 Universitätszahnklinik Wien GmbH (School of Dentistry Vienna), Sensengasse 2a, 1090 Vienna, Austria; 3 RTI International, 3400 East Cornwallis Rd, Durham, NC 27740 USA

**Keywords:** Systematic review, Periodontal diseases, Periodontitis, Gingivitis, Screening, Treatment, Screening tools

## Abstract

**Background:**

Periodontal diseases are responsible for a vast burden of disease globally and are associated with other severe illnesses such as cardiovascular diseases or diabetes. Tests for early diagnosis of periodontal diseases and effective treatments are available. The effectiveness of screening for periodontal diseases to detect periodontal diseases at an early stage during periodic health examinations at primary care facilities, however, is unclear. The objective of this systematic review is to assess the benefits and risks of screening for periodontal diseases in adults during the periodic health examinations.

**Methods:**

We will use two methodological approaches: (1) a systematic review to assess the effectiveness and risk of harms of screening for periodontal diseases during periodic health examinations and (2) an overview of systematic reviews to determine the effectiveness of treatment approaches for early periodontal disease. We will search electronic databases (Ovid MEDLINE, Embase.com, the Cochrane Library, Epistemonikos, Centre for Reviews and Dissemination databases, PubMed (non-MEDLINE content)) for published studies as well as sources for grey literature to detect unpublished studies. Two authors will independently screen abstracts and full texts using pre-defined eligibility criteria, select studies, extract data, and assess the risk of bias of included studies or reviews. In general, we will conduct a systematic narrative synthesis. Criteria for conducting meta-analyses were defined a priori. Our primary outcomes of interest are tooth loss, loosening of teeth, and depletion of bone tissue. Secondary outcomes are gingivitis/gum inflammation, pocket depths, dental hygiene, lifestyle modifications (e.g., smoking, alcohol, nutrition), and toothache. We consulted a panel of experts and patient representatives to prioritize these outcomes. Two investigators will assess independently the certainty of the evidence for each outcome using the GRADE (Grading of Recommendations Assessment, Development and Evaluation) approach.

**Discussion:**

We anticipate that our review will highlight the gaps in the available evidence about the effectiveness of screening for periodontal diseases during periodic health examinations. Implications for screening programs may be based on linked evidence about the validity of available screening tools and the effectiveness of early treatment.

**Systematic review registration:**

PROSPERO CRD42017081150

**Electronic supplementary material:**

The online version of this article (10.1186/s13643-019-0977-9) contains supplementary material, which is available to authorized users.

## Background

### Rationale

Periodontal disease is defined as any disorder of the tissues surrounding and supporting the teeth. Commonly, the term periodontal disease refers to bacteria-induced inflammatory disorders of the periodontium [1]. Periodontal disease comprises the diseases gingivitis (the presence of gingival inflammation without loss of connective tissue) and periodontitis (inflammatory disease that encompasses the supporting tissues, i.e., the connective tissue and the bone around the teeth) [2, 3]. Several different forms of gingivitis and periodontitis exist; plaque-induced gingivitis and chronic periodontitis are the most common forms [4].

In 2010, severe periodontitis was the sixth most prevalent condition globally, affecting 11% of the world’s population [5]. According to the Global Burden of Disease Study 2015, periodontal diseases were responsible for 3.5 million years lived with disease or disability worldwide (95% CI, 1.4 to 7.3 million) [6]. Besides unpleasant symptoms such as swelling and bleeding of the gums, halitosis, or pain, severe periodontitis can lead to loosening of teeth and tooth loss [1]. In fact, periodontal diseases are the leading cause for tooth loss [7]. In addition, periodontal diseases are also associated with increased risks for diabetes [8], cardiovascular disease [9], adverse pregnancy outcomes [10], and reduced quality of life [11].

Risk and prognostic factors for periodontal diseases include unmodifiable causes such as genetic determination or age, and modifiable factors such as smoking, nutritional habits [12], and the presence of putative periodontal pathogens [3].

Early interventions to treat periodontal disease focus on diminishing inflammation by plaque control, counseling on oral hygiene, and mechanical debridement (e.g., scaling and root planing). The most important and cost-effective intervention against plaque-induced periodontal diseases is brushing teeth which mechanically removes about 50% of plaque [13]. Although repeated counseling by dental professionals increases patient knowledge about oral hygiene in adults, findings are inconsistent concerning the effectiveness of counseling on the reduction of gingivitis [14]. The most common treatment for periodontal diseases is quadrant-wise scaling and root planing [15]. Scaling refers to the removal of plaque, calculus, and stain from teeth; root planing is a procedure to remove diseased cementum and/or dentin [16]. The evidence is insufficient to determine whether local antibiotics or anti-inflammatory drugs [17], as well as full-mouth scaling or full-mouth disinfection [15], provide additional clinical benefits to quadrant scaling and root planing.

If periodontal disease is advanced or if initial treatment is not sufficient, dentists can make adjunctive use of either systemic antibiotics [18] or various types of periodontal surgery [1] for additional clinical improvement of probing pocket depths, probing attachment levels, or bone levels.

Periodic health examinations are an important element of primary health care in many countries. They consist of counseling activities and various screening tests to detect risk factors or diseases at an early stage in asymptomatic people [19]. The overall goal of periodic health examinations is to reduce morbidity and mortality, which requires the availability of effective treatments for screen-detected risk factors or early stage diseases [20]. Despite effective early treatment options, to date, it remains unclear whether screening for periodontal diseases during periodic health examinations by non-dental health professionals leads to a reduction of the morbidity of periodontal diseases.

Several methods are available for the diagnosis of periodontal diseases, such as measuring pocket depths via periodontal probes, ultrasonographic probes, X-rays, or cone beam computed tomography [21–24]. However, most diagnostic tests require special equipment and training and are not feasible as screening tools during periodic health examinations by non-dental health professionals.

Screening approaches for periodontal diseases by non-dental health professionals include inspection of the oral cavity or the use of self-reported questionnaires which assign risk scores [25–27]. Based on such risk scores, physicians or other non-dental health professionals can provide counseling about dental hygiene and modifiable risk factors or refer patients to dentists for further assessment [19]. International guidelines [28–31] currently do not provide recommendations for or against screening for periodontal diseases during periodic health examinations.

The objective of this systematic review is to assess the published and unpublished literature to determine the benefits and risks of screening for periodontal diseases in adults during periodic health examinations by non-dental health professionals.

### Objectives

Our review will be guided by five key questions (KQ):

- *KQ 1*: What are the benefits of screening for periodontal diseases during periodic health examinations by non-dental health professionals in adults aged 18 years or older compared with no screening?
- *KQ 2*: What are potential harms of screening for periodontal diseases during periodic health examinations by non-dental health professionals in adults aged 18 years or older compared with no screening?
- *KQ 3*: What are valid and reliable screening tools for periodontal disease that can be used during periodic health examinations?
- *KQ 4*: In patients with a diagnosis of periodontal disease, what is the effectiveness of early treatment to improve health outcomes?
- *KQ 5*: What are potential harms of early treatment of periodontal diseases?

**Fig. 1:**
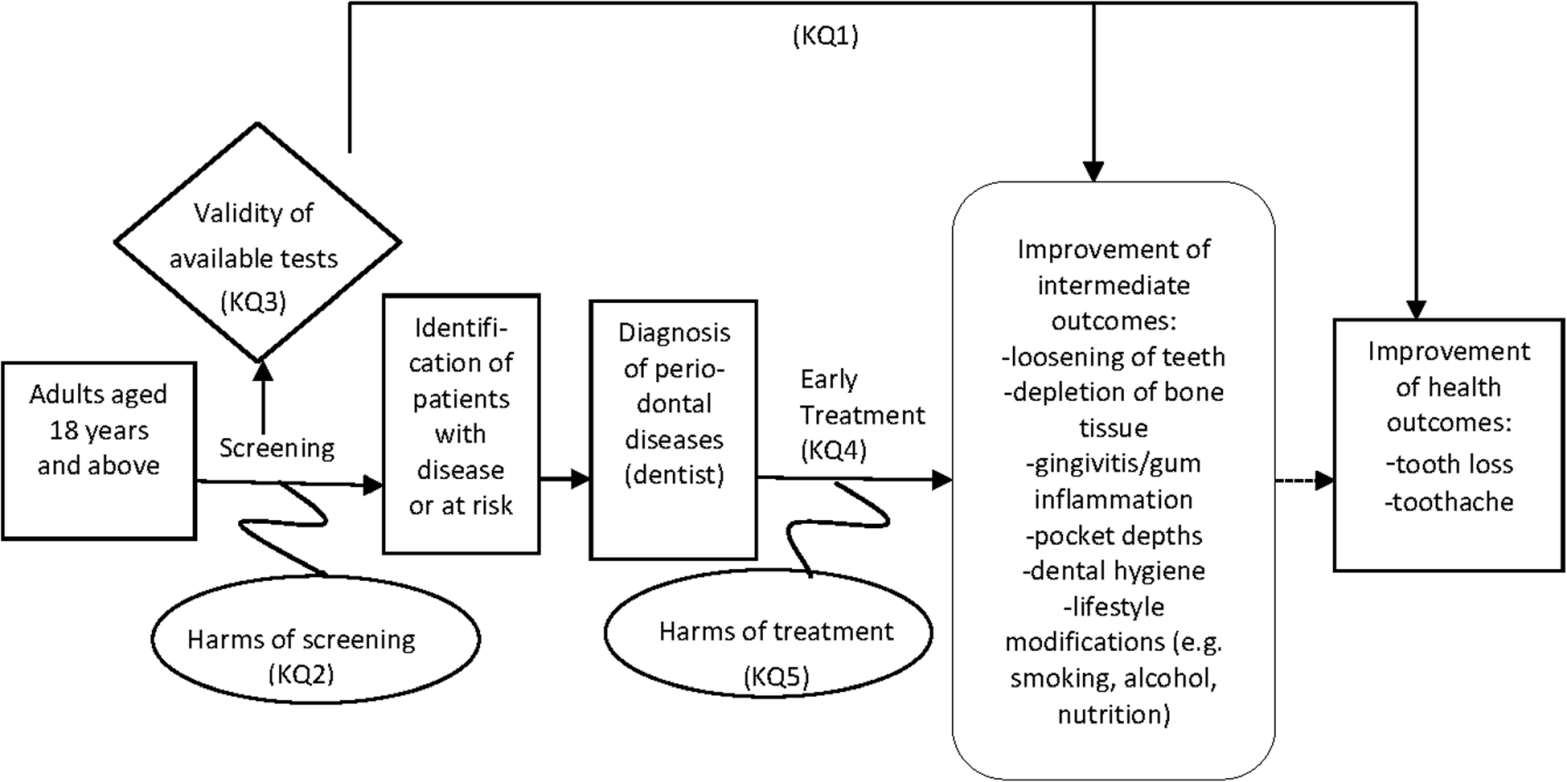
Analytic framework for screening for periodontal diseases during periodic health examinations

Figure 1 presents an analytic framework of the effects of screening for periodontal diseases during periodic health examinations on relevant health outcomes.

## Methods

We have registered the protocol of the systematic review with the International Prospective Register of Systematic Reviews (PROSPERO), registration number CRD42017081150. To address our key questions of interest, we will employ two different methodological approaches. For KQs 1 to 3, we will conduct a systematic literature review. For KQs 4 and 5, we will employ an overview of systematic reviews. The following sections present the two approaches in more details. Throughout the protocol, we follow the Preferred Reporting Items for Systematic Review and Meta-Analysis Protocols (PRISMA-P) statement [32].

### Systematic review (KQs 1–3)

We will employ a systematic review of primary studies to determine the effects and potential harms of mass screening for periodontal diseases (KQs 1 and 2) as well as the validity of screening tools (KQ 3) that could be used during periodic health examinations.

#### Eligibility criteria

Inclusion and exclusion criteria are listed in Table 1 and described in more details below.

**Table 1:** Inclusion and exclusion criteria systematic review

	Inclusion	Exclusion
Study design	- Randomized and non-randomized controlled trials - Diagnostic studies	Systematic reviews All other study designs
Population	Adults aged 18 years and above without known periodontal disease	People with known periodontal disease Children, pregnant women, populations with increased risks for periodontal diseases
Interventions	Screening for periodontal diseases by non-dental health professionals using methods that could be applied during periodic health examinations without specialized dental equipment Examples: self-reported questionnaires, inspection of the oral cavity, blood sampling, saliva sampling	Screening for periodontal diseases using methods that require special dental training or specialized dental equipment
Comparison	KQ 1 and KQ 2: no screening for periodontal disease KQ 3: validated diagnosis of periodontal disease by a dentist	KQ 1 and KQ 2: other screening tests
Outcomes	No limitations	None
Setting	KQ 1 and KQ 2: periodic health examination in a primary care setting KQ 3: no limitations	KQ 1 and KQ 2: dentists, studies conducted outside office-based primary care settings, e.g., homecare or mobile examination units
Country	Countries having a very high UNESCO human development index: Andorra, Argentina, Australia, Austria, Bahrain, Belgium, Brunei Darussalam, Canada, Chile, Croatia, Cyprus, Czech Republic, Denmark, Estonia, Finland, France, Germany, Greece, Hong Kong China (SAR), Hungary, Iceland, Ireland, Israel, Italy, Japan, Korea (Republic of Korea), Kuwait, Latvia, Liechtenstein, Lithuania, Luxembourg, Malta, Montenegro, Netherlands, New Zealand, Norway, Poland, Portugal, Qatar, Romania, Russian Federation, Saudi Arabia, Singapore, Slovakia, Slovenia, Spain, Sweden, Switzerland, United Arab Emirates, UK, USA	All other countries
Publication language	English, German	Non-English or non-German language

*KQ* key question, *UNESCO* United Nations Educational, Scientific and Cultural Organization

##### Study designs

We will include randomized controlled trials and non-randomized controlled trials, and diagnostic studies. We will exclude any other study designs, systematic reviews, non-systematic reviews, letters, commentaries, and editorials.

##### Participants

For studies addressing the benefits and harms of screening, our target population is adults aged 18 years and older without known periodontal diseases. We will exclude studies in children or pregnant women and studies conducted exclusively in populations with increased risks for periodontal diseases. We will not limit our target population by medical conditions, co-existent conditions, ethnicity, socio-economic status, or further participant-related characteristics.

##### Interventions

We will include eligible studies that assess the effectiveness of screening approaches for periodontal diseases that are feasible in non-dental primary care settings during periodic health examinations by non-dental health professionals such as self-reported questionnaires, inspection of the oral cavity, blood or saliva sampling, or others. We will exclude interventions that require special training or specialized dental equipment. We will not apply any restrictions on the duration of the intervention.

##### Comparators

Control interventions are “no screening interventions” for KQs 1 and 2. For KQ 3, we will use a confirmed diagnosis of periodontal disease by a dentist as the reference test.

##### Outcomes

No eligibility criteria will be set under this section.

##### Timing

We will not apply any restrictions on eligible time points for measuring outcomes.

##### Settings

Settings of interest for KQ 1 and KQ 2 include all office-based primary care settings. We will exclude studies conducted at dental practitioner offices or dental clinics for KQ 1 and KQ 2. We will also exclude studies conducted outside office-based primary care settings, e.g., homecare or mobile examination units. For KQ 3, we will include all types of settings without limitations.

##### Countries

We will include all studies conducted in countries having a very high UNESCO (United Nations Educational, Scientific and Cultural Organization) human development index [32]. We will exclude studies conducted in all other countries.

##### Language

We will consider only publications in English or German languages.

#### Information sources and search strategy

We will search the following electronic databases: Ovid MEDLINE, the Cochrane Library, Embase, and PubMed (non-MEDLINE content).

The searches will consider publications from January 2007 through the search date. Due to financial and time constraints, we will not consider publications published before 2007. We will first develop a search strategy for Ovid MEDLINE and will then translate it to fit other electronic databases. We will consider only publications limited to “humans.” An experienced information specialist will perform all searches in collaboration with a dental health professional. According to the PRESS (Peer Review of the Electronic Search Strategy) statement [33], the electronic Ovid MEDLINE search strategy will be peer-reviewed by a second information specialist.

In addition, we will search for grey literature (i.e., unpublished studies) relevant to this review. Potential sources of grey literature include ClinicalTrials.gov, the World Health Organization’s International Clinical Trials Registry Platform, Google Scholar, and dissertation databases (e.g., DART-Europe).

To avoid retrieval bias, we will manually search the reference lists of background articles on this topic to look for any relevant citations that our electronic searches might have missed. If our search retrieves conference abstracts about studies that might meet our inclusion criteria, we will manually search for further information about these studies (e.g., publications, entries in trial registries).

#### Study records

##### Data management

Identified citations will be stored in an EndNote® X8 bibliographic database (Thomson Reuters, New York, NY, USA). All results of the abstract and full-text review including information on the reasons for exclusion during full-text review will be recorded in the EndNote database.

Pdf files of all full-text articles will be stored on a server that is accessible for all members of the review team.

##### Selection process

We will develop and pilot test abstract and full-text review forms that reflect our inclusion and exclusion criteria. Two reviewers will independently screen abstracts and full-text articles and assess their eligibility. We will divide screening work between BT, BNS, LA, and CK. Any discrepancies will be resolved through discussion or consultation with a third reviewer. If uncertainties about the eligibility of an article remain, we will contact the article authors. Abstract and full-text reviews will be carried out with Covidence (https://www.covidence.org/). A total of 50 abstracts will be piloted by all reviewers to calibrate reviewers, fine-tune eligibility criteria, and test the abstract review form. Full-text review will be piloted with 10 full-text articles. The excluded studies and the rationale for their exclusion will be given in an additional file to the completed review.

##### Data collection process

We designed a structured data abstraction form. After the screening process is finished, we will pilot test the form. Additional file 1 presents the pre-specified data abstraction form. Due to limited resources, the data will be extracted by one reviewer and checked for completeness and accuracy by a second investigator. We will divide data extraction work between BT, BNS, LA, and CK. The data extraction process will be piloted with one study.

##### Data items

For studies that meet our inclusion criteria on KQ 1 or KQ 2, we will abstract the following information: (a) author, title, year of publication; (b) population (overall number of participants, number of analyzed participants, country, mean age, percent female, percent white); (c) screening intervention (characteristics of screening intervention, duration, tools used); (d) control intervention; (e) outcomes and outcome measures (what outcomes were measured and how they were measured); (f) results (presented as dichotomous or continuous outcome measures with 95% confidence intervals); and (g) funding source.

For studies that meet our inclusion criteria on KQ 3, we will abstract the following information: (a) author, title, year of publication; (b) population (overall number of participants, number of analyzed participants, country, mean age, percent female, percent white); (c) screening intervention (index test); (d) control intervention (reference test); (e) outcomes (sensitivity, specificity, positive predictive value, negative predictive value, other validity data/other important information about validity); and (f) funding source.

We will contact study authors if relevant data are not reported in an included publication.

#### Outcomes and prioritization

To prioritize outcomes, we followed guidance of the GRADE (Grading of Recommendations Assessment, Development and Evaluation) Working Group [34]. Clinical experts and patient representatives rated the relative importance of outcomes on a Likert scale from 1 (not relevant) to 9 (critical) via a web-based survey using a modified Delphi approach [35]. We considered only the eight highest-ranked outcomes. Clinical experts and patient representatives ranked the following outcomes as critical (outcomes with mean number of points at 9-item Likert scale): tooth loss (7.4), loosening of teeth (7.25), and depletion of bone tissue (7.1). We will consider these critical outcomes as primary outcomes. Outcomes ranked as important but not critical are gingivitis/gum inflammation (6.86), pocket depths (6.24), dental hygiene (6.1), lifestyle modifications (e.g., smoking, alcohol, nutrition; 6.05), and toothache (6.05). We will consider these outcomes as secondary outcomes.

We plan to calculate outcome measures such as risk ratios or mean differences. For example, for the risk ratio of tooth loss, we will calculate the risk of tooth loss in participants screened by non-dental health professionals relative to the risk of tooth loss in participants that were not screened by non-dental health professionals. For example, for the mean difference of tooth loss, we will calculate the mean difference in the number of lost teeth between participants screened by non-dental health professionals and those that were not screened by non-dental health professionals.

All statistical analyses will be based on the abovementioned outcome measures. For diagnostic studies, we will consider all outcomes addressing diagnostic test accuracy.

For KQ 1, we will consider studies with at least 3 months of follow-up. For KQ 2, we will not apply any restrictions on eligible time points for measuring potential harms as they can appear immediately after an intervention. For KQ 3, we will only consider studies not exceeding 2 weeks between conducting the index test and the reference test.

#### Risk of bias in research studies

Risk of bias will be assessed by two independent reviewers using the Cochrane Risk of Bias Tool 2 [36] for randomized controlled trials and the ROBINS-I (Risk Of Bias In Non-randomized Studies - of Interventions) tool [37] for non-randomized controlled trials. For diagnostic studies, we will use the QUADAS-2 (Quality Assessment of Diagnostic Accuracy Studies) tool [38]. We will consider the risk of bias for each relevant outcome of a study. Disagreements between reviewers will be resolved by discussion and consensus or by consulting a third reviewer.

#### Data synthesis

##### Criteria for a quantitative synthesis

We will base decisions on whether or not to conduct quantitative syntheses (i.e., meta-analyses) of included studies on recommendations from the US Agency for Healthcare Research and Quality Evidence-based Practice Centers (AHRQ) [39]. The AHRQ “pooling decision tree” takes clinical and methodological heterogeneity, the risk for misleading results (e.g., through small studies effects), the number of studies to be pooled, statistical heterogeneity, and the presence of large or best-quality trials into consideration.

If we detect three or more studies that we deem to be similar enough regarding population, interventions, and comparators, we will conduct meta-analyses. We will include high risk of bias studies for sensitivity analyses only.

We will consider performing pairwise meta-analyses with at least three unique studies of low or medium risk of bias that we deem to be sufficiently similar (in population, interventions, comparators, and outcomes). We will include high risk of bias studies for sensitivity analyses only. For each meta-analysis, we will carefully examine differences in populations, interventions, and other clinically relevant factors to determine clinical heterogeneity among studies. We will use the DerSimonian and Laird random-effects model or the Mantel-Haenszel fixed-effects model, depending on the underlying clinical heterogeneity and the clinical relevance of the assumptions that characterize each approach. For meta-analyses of non-randomized studies, we will use generic inverse variance models to combine effects of individual studies that are adjusted for potential confounders.

We do not plan to conduct meta-analyses on diagnostic test accuracy studies. A diagnostic test accuracy meta-analysis requires at least five studies with the same index and reference tests over a similar follow-up time. We do not expect that we can meet these thresholds based on preliminary scoping searches and the available literature. In addition, we expect a wide range of possible index tests (e.g., various questionnaires or saliva tests) which could not be pooled because small differences, e.g., in items of the questionnaire, can change the test accuracy.

For all analyses, we will use Review Manager 5.3 [40].

##### Summary measures

We will determine dichotomous data by using risk ratio (RR) with 95% confidence interval (CI) and continuous outcomes using weighted mean differences (with 95% CI) or standardized mean differences (95% CI) if different measurement scales are used.

##### Unit of analysis

Because we are not including cluster-randomized trials, the unit of analysis is the individual study participant.

##### Missing data

We will take the following steps to deal with relevant missing data:

- Contact the authors of the included studies via email or phone;
- Screen the study and investigate important numerical data such as randomized individuals as well as intention-to-treat, as-treated, and per protocol populations;
- Investigate attrition rates as part of the “risk of bias” assessment in terms of drop-outs, losses to follow-up and withdrawals;
- Critically appraise issues of missing data and imputation methods (e.g., last observation carried forward);
- Impute missing standard deviations if contacted authors do not respond;
- Apply sensitivity analyses to estimate the impact of imputation on meta-analyses.

##### Assessment of heterogeneity

To assess statistical heterogeneity in effects between studies, we will calculate the chi-squared statistic and the *I*^2^ statistic (the proportion of variation in study estimates attributable to heterogeneity rather than due to chance) [41, 42]. For the chi-squared statistic, we will adopt a *p* value of 0.1 as a threshold for clinical significance. In cases of high heterogeneity, we will explore potential reasons for heterogeneity. If we encounter high unexplained heterogeneity, we will not perform any quantitative syntheses.

##### Additional analyses

We will exclude non-randomized studies with high risk of bias or studies that are unadjusted for potential confounders for our data synthesis, but we will include them for sensitivity analyses.

We do not plan any subgroup analyses as part of the general data analyses. If meta-analyses have high heterogeneity and meta-regression is not possible, we will conduct subgroup analyses to explore heterogeneity using the following variables:

- Age younger than 65 years versus 65 years and older
- Family physicians versus general internists
- Female versus male participants

##### Narrative synthesis

Irrespective of whether or not we could perform meta-analysis, we will present characteristics and results of included studies of any level of risk of bias in tables and synthesize data narratively. We will present results by key question. Within key question, we will first present main outcomes then secondary outcomes as presented under eligibility criteria.

#### Meta-bias

To assess potential publication bias, we will use funnel plots, Egger’s regression [43], and Begg-Mazumdar [44] correlation in meta-analyses of 10 or more studies. If we suspect publication bias, we will consider this fact in our interpretation of results. We will evaluate whether selective reporting of outcomes is present by comparing included studies with published protocols, if available. If we suspect selective reporting in trials without published protocols, we will contact study authors.

#### Confidence in cumulative evidence

We will grade the certainty of evidence for each outcome that the panel deemed to be critical or important for decision-making. We will dually assess the certainty of evidence for outcomes of interest using the GRADE approach [45, 46]. Discrepancies will be resolved through discussion or consultation with a third reviewer. The certainty of evidence reflects the extent to which we are confident that an estimate of the effect is correct. Table 2 presents grades of certainty of evidence and their definitions.

**Table 2:** Grades of evidence according to GRADE and their definitions

Grade	Definition
High	We are very confident that the true effect lies close to that of the estimate of the effect.
Moderate	We are moderately confident in the effect estimate: The true effect is likely to be close to the estimate of the effect, but there is a possibility that it is substantially different.
Low	Our confidence in the effect estimate is limited: The true effect may be substantially different from the estimate of the effect.
Very Low	We have very little confidence in the effect estimate: The true effect is likely to be substantially different from the estimate of effect.

### Overview of reviews (KQs 4-5)

Because preliminary searches revealed sufficient evidence on effectiveness of treatment options for periodontal disease, we will conduct an overview of systematic reviews to assess the effects and potential harms of early treatment (non-surgical treatment) for periodontal diseases. Our aim for this overview of reviews is to summarize evidence from more than one systematic review of different interventions for the same condition or problem as described in the Cochrane Handbook [47]. Overviews of reviews are a relatively new methodological approach and some aspects of the methodology remain uncertain [48]. We consulted methodological papers on overviews of reviews to determine the following methods [48–53]. Because overviews of systematic reviews are a new and developing methodological field, we will implement relevant methodological developments during the conduct of the overview. We will document and report any modifications with the respective rationale.

#### Eligibility criteria

Inclusion and exclusion criteria are listed in Table 3 and described in more details below.

**Table 3:** Inclusion and exclusion criteria overview of reviews

	Inclusion	Exclusion
Study design	Systematic reviews with or without meta-analyses	All other study designs
Population	Adults aged 18 years and above with diagnosed periodontal disease	People without diagnosed periodontal disease Children, pregnant women, populations with increased risks for periodontal diseases
Interventions	Early treatments for periodontal diseases (non-surgical treatments)	Surgical treatments
Comparison	No treatment or other early (non-surgical) treatments	Surgical treatments
Outcomes	No limitations	None
Setting	No limitations	
Country	At least 50% of included studies have to be conducted in countries having a very high UNESCO human development index: Andorra, Argentina, Australia, Austria, Bahrain, Belgium, Brunei Darussalam, Canada, Chile, Croatia, Cyprus, Czech Republic, Denmark, Estonia, Finland, France, Germany, Greece, Hong Kong China (SAR), Hungary, Iceland, Ireland, Israel, Italy, Japan, Korea (Republic of Korea), Kuwait, Latvia, Liechtenstein, Lithuania, Luxembourg, Malta, Montenegro, Netherlands, New Zealand, Norway, Poland, Portugal, Qatar, Romania, Russian Federation, Saudi Arabia, Singapore, Slovakia, Slovenia, Spain, Sweden, Switzerland, United Arab Emirates, UK, USA If countries of primary studies are not reported in the systematic review we will include this reference and report on doubts about the applicability to countries having a very high UNESCO human development index.	All other countries
Date of search	1/2007 onwards	Before 1/2007
Publication language	English, German	Non-English or non-German language

*KQ* key question, *UNESCO* United Nations Educational, Scientific and Cultural Organization

##### Study designs

For effectiveness and harms of treatment, we will include only systematic reviews of randomized and non-randomized controlled trials. Systematic reviews are defined based on the Cochrane handbook [54] as a literature review that attempts to collate all empirical evidence using (a) clearly stated objectives and pre-defined eligibility criteria, (b) an explicit reproducible methodology, (c) a systematic search, (d) an assessment of the validity of the findings of the included studies, and (e) a systematic presentation, and synthesis, of the characteristics and findings of the included studies. We will exclude any other study designs, non-systematic reviews, letters, commentaries, and editorials.

##### Participants

Systematic reviews that addressed treatments of periodontal disease in adults aged 18 years or older with diagnosed periodontal disease will be eligible. Systematic reviews that included children, pregnant women, or populations with increased risks for periodontal disease will not be eligible. We will not limit our target population by medical condition, co-existent conditions, ethnicity, socio-economic status, or other participant-related characteristics.

##### Interventions

Because our aim for this overview of reviews is to summarize evidence of different interventions for the same condition, we will include systematic reviews examining any non-surgical, evidence-based therapy that is used to treat periodontal diseases.

##### Comparators

We will include systematic reviews that used “no active treatment” as a comparator as well as other early (non-surgical) treatments to examine comparative effectiveness.

##### Outcomes

To prioritize outcomes, we followed guidance of the GRADE (Grading of Recommendations Assessment, Development and Evaluation) Working Group [34] as presented above in the protocol for the systematic review.

Our primary outcomes are those that citizens and experts ranked as critical for decision-making: tooth loss, loosening of teeth, and depletion of bone tissue. The following outcomes that citizens and experts ranked as important but not critical for decision-making are secondary outcomes: gingivitis/gum inflammation, pocket depths, dental hygiene, lifestyle modifications (e.g., smoking, alcohol, nutrition), and toothache.

We will include systematic reviews that report any of the abovementioned outcomes, regardless of whether these outcomes were primary or secondary outcomes of the systematic reviews.

##### Timing

We will not exclude systematic reviews because of restrictions on eligible time points for measuring outcomes.

##### Settings

We will include all relevant systematic reviews regardless of restrictions to settings that the reviews had applied.

##### Countries

We will include all systematic reviews where at least half of the included studies were conducted in countries with a very high UNESCO (United Nations Educational, Scientific and Cultural Organization) human development index [32]. If countries of primary studies are not reported in the systematic review, we will include this reference but document the potential lack of applicability to countries with a very high UNESCO human development index.

##### Language

We will consider only publications in English or German languages.

#### Information sources and literature search

We will search the following electronic databases: Ovid MEDLINE, the Cochrane Library, PubMed (non-MEDLINE content), Epistemonikos, and CRD (Centre for Reviews and Dissemination) databases.

The search will consider publications from January 2007 through the search date. We will not consider systematic reviews before 2007 because they are out of date [49]. We will first develop a search strategy for Ovid MEDLINE and will then translate it to fit other electronic databases. We will consider only publications limited to “humans.” An information specialist with expertise in systematic searches of the literature will conduct all literature searches in collaboration with a dental health professional. According to the PRESS (Peer Review of the Electronic Search Strategy) statement [33], the electronic Ovid MEDLINE search strategy will be peer-reviewed by a second information specialist (Additional file 2).

To avoid retrieval bias, we will manually search the reference lists of background articles on this topic to look for any relevant citations that our electronic searches might have missed. If our search retrieves conference abstracts about studies that might meet our inclusion criteria, we will manually search for further information about these studies (e.g., publications, entries in trial registries).

#### Study records

##### Data management, selection process, and data collection process

We will use the same data management, selection process, and data collection process as presented for systematic reviews.

##### Data items

For studies that meet our inclusion criteria on KQ 4 or KQ 5, we will abstract the following information: (a) author, title, and year of publication; (b) date of search; (c) country (of included studies); (d) objective of review; (e) types of studies included in review; (f) participants included in review; (g) intervention included in review (brief description); (h) comparisons included in review; (i) outcomes included in review; (j) target condition being addressed in the review; (k) number of studies included in review; (l) number of participants included in review; and (m) outcomes (effect sizes, confidence intervals, heterogeneity, direction of effect) [49].

#### Outcomes and prioritization

Our primary outcomes of interest are tooth loss, loosening of teeth, and depletion of bone tissue. Secondary outcomes are gingivitis/gum inflammation, pocket depths, dental hygiene, lifestyle modifications (e.g., smoking, alcohol, nutrition), and toothache. We will also consider any kind of harms as outcomes of interest. For KQ 4 and KQ 5, we will not apply any restrictions on eligible time points for measuring outcomes. We will report limitations on time points for measuring outcomes that were set in the included systematic reviews.

#### Risk of bias in research studies

For the appraisal of systematic reviews, we will use the ROBIS (Risk of Bias in Systematic Reviews) tool [55]. ROBIS was designed following the most recent methods for developing risk of bias tools and is used specifically to assess the risk of bias of systematic reviews. ROBIS was already used by other authors of overviews of systematic reviews [49], and a comparison to other tools is not available yet [56]. We will consider the risk of bias for each relevant outcome of a study. Additionally, we will use AMSTAR 2 (A MeaSurement Tool to Assess systematic Reviews) [57] for the identification of high-quality systematic reviews.

Two reviewers will independently appraise the risk of bias of included systematic reviews. Disagreements between reviewers will be resolved by discussion and consensus or by consulting a third reviewer. AMSTAR 2 scores for each included systematic review will be uploaded as an additional file in the completed overview of reviews.

#### Data synthesis

Our aim for this overview of reviews is to summarize evidence from more than one systematic review of different interventions (various early treatments) for the same condition or problem as described in the Cochrane Handbook [47]. As preliminary searches revealed sufficient evidence on effectiveness of various options of early treatment of periodontal disease, we will apply a best evidence synthesis approach [58]. We will categorize all included studies via type of treatment and will only synthesize data from the most recent review with low risk of bias in every category narratively. This methodological approach allows us to overcome the challenge of overlap between reviews if studies appear in more than one review [50] as well as the challenge of differences in the methodological quality of included reviews [49].

#### Meta-bias

To avoid meta-bias, we will consider only the most reliable evidence, using a best evidence synthesis approach [58].

#### Confidence in cumulative evidence

When authors of included systematic reviews report on certainty of evidence, we will adopt this judgment for comparisons of different treatments. If the certainty of evidence is not reported in included systematic reviews, we will grade the certainty of evidence as presented for systematic reviews.

## Differences between the protocol and the review

If changes of methods will be necessary, we will fully document them and present them in the completed reviews. We will point out any methods that were changed to this protocol, summarize methods that could not be implemented, explain any changes in methods from this protocol, and provide the rationales for the changes as described in the Cochrane Handbook [47].

## Discussion

The methodological approach of our study which we outline in this protocol has several limitations. First, we will limit the searches to studies published in English or German languages published within the past 12 years. Second, for efficiency reasons, we employ an overview of reviews to determine the effectiveness of treatments for early-stage periodontal disease. Overviews of reviews are a relatively new methodological approach that has limitations such as the reliance on decisions that other systematic review authors had made. Although we will assess the methodological soundness of eligible systematic reviews, overviews of reviews have a larger degree of uncertainty than de novo systematic reviews. Third, due to limited resources, we have chosen that data will be extracted by one reviewer and checked for completeness and accuracy by a second investigator rather than two reviewers will extract data independently. To the best of our knowledge, this will be the first systematic review to assess the potential benefits and harms of screening for periodontal diseases during periodic health examinations by non-dental health professionals in adults aged 18 and older. If we find little or no direct evidence addressing the benefits and risks of screening, we will try to answer the key questions via linked evidence. Regardless of our findings, our systematic review will provide an objective base for health policy decision-makers and guideline developers to recommend for or against screening for periodontal diseases during periodic health examinations in primary care settings.

## Additional files

Additional file 1: Data abstraction form. (XLSX 18 kb)

Additional file 2: Draft MEDLINE search strategy. (DOCX 18 kb)

## Abbreviations

BNS: Barbara Nussbaumer-Streit
BT: Birgit Teufer
CI: Confidence interval
CK: Christina Kien
CRD: Centre for Reviews and Dissemination
GRADE: Grading of Recommendations Assessment, Development and Evaluation
KQ: Key question
LA: Lisa Affengruber
PRESS: Peer Review of the Electronic Search Strategy
PRISMA-P: Preferred Reporting Items for Systematic Reviews and Meta-Analysis Protocols
PROSPERO: International Prospective Register of Systematic Reviews
QUADAS-2: Quality Assessment of Diagnostic Accuracy Studies 2
ROBINS-I: Risk Of Bias In Non-randomized Studies - of Interventions
ROBIS: Risk of Bias in Systematic Reviews
UNESCO: United Nations Educational, Scientific and Cultural Organization
