# Supplementary material for: Screening for periodontal diseases by non-dental health professionals: a protocol for a systematic review and overview of reviews
Source: Syst Rev. 2019 Feb 25;8:61. doi: 10.1186/s13643-019-0977-9 (PMC6388477; doi:10.1186/s13643-019-0977-9)
Supplement: Supplementary file 2 — Draft MEDLINE search strategy. (DOCX 18 kb) [file 13643_2019_977_MOESM2_ESM.docx]

**Draft MEDLINE search strategy**

**KQs 1, 2 & 3**

| Ovid MEDLINE(R) 1946 to November Week 2 2017, Ovid MEDLINE(R) Epub Ahead of Print November 15, 2017, Ovid MEDLINE(R) In-Process & Other Non-Indexed Citations November 15, 2017, Ovid MEDLINE(R) Daily Update November 15, 2017 | | |
| --- | --- | --- |
| 17.Nov.17 |  |  |
| **#** | **Searches** | **Results** |
| 1 | periodontal diseases/ or exp gingivitis/ or exp periodontitis/ | 58394 |
| 2 | periodontitis.ti,ab,kf. | 23031 |
| 3 | gingivitis.ti,ab,kf. | 7335 |
| 4 | ((periodont* or parodont* or gingiv* or gum?) adj3 (disease* or inflamm* or bleeding or defect*)).ti,ab,kf. | 31894 |
| 5 | or/1-4 | 74353 |
| 6 | Mass Screening/ | 99186 |
| 7 | Multiphasic Screening/ | 1150 |
| 8 | Early Diagnosis/ | 24154 |
| 9 | Preventive Health Services/ | 12967 |
| 10 | Diagnostic Tests, Routine/ | 10438 |
| 11 | screen*.ti. | 160331 |
| 12 | Screening?.ab,kf. | 419091 |
| 13 | health check?.ti,ab,kf. | 3337 |
| 14 | ((periodic* or routin* or preventiv* or annual* or regular* or year*) adj3 (examination* or check? or screen* or test*)).ti,ab,kf. | 95785 |
| 15 | (early adj (detection or diagnosis or identification)).ti,ab,kf. | 135906 |
| 16 | *Health Promotion/ | 45412 |
| 17 | *Primary Prevention/ | 8500 |
| 18 | "surveys and questionnaires"/ or self report/ | 435921 |
| 19 | (questionnaire? or self report*).ti,ab,kf. | 543082 |
| 20 | Diagnosis, Oral/ | 1852 |
| 21 | ((oral or mouth or buccal) adj2 (exam* or screen*)).ti,ab,kf. | 6616 |
| 22 | or/6-21 | 1462272 |
| 23 | 5 and 22 | 5296 |
| 24 | exp animals/ not exp humans/ | 4743082 |
| 25 | 23 not 24 | 5194 |
| 26 | exp age groups/ not exp adult/ | 1851515 |
| 27 | 25 not 26 | 4646 |
| 28 | (english or german).lg. | 25879274 |
| 29 | 27 and 28 | 4318 |
| 30 | limit 29 to yr="2007 -Current" | 2687 |
| 31 | remove duplicates from 30 | 2538 |
| 32 | exp *Pregnancy/ | 141664 |
| 33 | *Prenatal Diagnosis/ or *Prenatal Injuries/ or *Prenatal Care/ or *Prenatal Exposure Delayed Effects/ or *Prenatal Education/ | 55559 |
| 34 | (pregnan* or prenatal*).ti. | 259811 |
| 35 | case report/ | 2002447 |
| 36 | ((case? and (report* or series)) or "a case").ti. | 539346 |
| 37 | or/32-36 | 2535356 |
| 38 | 31 not 37 | 2311 |

**KQs 4 & 5**

| Ovid MEDLINE(R) 1946 to November Week 2 2017, Ovid MEDLINE(R) Epub Ahead of Print November 17, 2017, Ovid MEDLINE(R) In-Process & Other Non-Indexed Citations November 17, 2017, Ovid MEDLINE(R) Daily Update November 17, 2017 | | |
| --- | --- | --- |
| 20.Nov.17 |  |  |
| **#** | **Searches** | **Results** |
| 1 | periodontal diseases/ or exp gingivitis/ or exp periodontitis/ | 58394 |
| 2 | periodontitis.ti,ab,kf. | 23039 |
| 3 | gingivitis.ti,ab,kf. | 7336 |
| 4 | ((periodont* or parodont* or gingiv* or gum?) adj3 (disease* or inflamm* or bleeding or defect*)).ti,ab,kf. | 31901 |
| 5 | or/1-4 | 74364 |
| 6 | exp oral hygiene/ or oral hygiene.ti,ab,kf. or dental hygiene.ti,ab,kf. | 26800 |
| 7 | exp Dental Prophylaxis/ or ((oral or dental) adj prophyla*).ti,ab,kf. | 8196 |
| 8 | Counseling/ | 34640 |
| 9 | exp anti-infective agents/ | 1574132 |
| 10 | exp Anti-Inflammatory Agents/ | 500031 |
| 11 | Dental Plaque/pc, th, dt | 5718 |
| 12 | ((early or nonsurg* or non surg*) adj2 (treat* or therap* or intervent*)).ti,ab,kf. | 88987 |
| 13 | (((root or dental or subgingiv* or supragingiv*) adj2 (scaling* or planing*)) or (periodontal adj3 debridement*)).ti,ab,kf. | 3085 |
| 14 | (toothbrush* or ((tooth or teeth) adj2 brush*)).ti,ab,kf. | 7103 |
| 15 | counsel?ing.ti,ab,kf. | 84646 |
| 16 | (antibiotic* or antibacterial* or anti bacterial* or anti inflamm* or antiinflamm*).ti,ab,kf. | 514746 |
| 17 | (plaque adj2 (remov* or control*)).ti,ab,kf. | 3370 |
| 18 | or/6-17 | 2431851 |
| 19 | 5 and 18 | 21425 |
| 20 | (systematic or structured or evidence or trials).ti. and ((review or overview or look or examination or update* or summary).ti. or review.pt.) | 131745 |
| 21 | (0266-4623 or 1469-493X or 1366-5278 or 1530-440X).is. | 16757 |
| 22 | meta-analysis.pt. or Network Meta-Analysis/ or (meta-analys* or meta analys* or metaanalys* or meta synth* or meta-synth* or metasynth*).tw,hw. | 161927 |
| 23 | review.pt. and ((medline or medlars or embase or pubmed or scisearch or psychinfo or psycinfo or psychlit or psyclit or cinahl or electronic database* or bibliographic database* or computeri#ed database* or online database* or pooling or pooled or mantel haenszel or peto or dersimonian or der simonian or fixed effect or ((hand adj2 search*) or (manual* adj2 search*))).tw,hw. or (retraction of publication or retracted publication).pt.) | 131911 |
| 24 | ((systematic or meta) adj2 (analys* or review)).ti,kf. or ((systematic* or quantitativ* or methodologic*) adj5 (review* or overview*)).tw,hw. or (quantitativ$ adj5 synthesis$).tw,hw. | 192011 |
| 25 | (integrative research review* or research integration).tw. or scoping review?.ti,kf. or (review.ti,kf,pt. and (trials as topic or studies as topic).hw.) or (evidence adj3 review*).ti,ab,kf. | 179977 |
| 26 | 20 or 21 or 22 or 23 or 24 or 25 | 449544 |
| 27 | 26 not (case report/ or letter.pt.) | 438091 |
| 28 | 19 and 27 | 769 |
| 29 | exp animals/ not exp humans/ | 4743082 |
| 30 | 28 not 29 | 767 |
| 31 | exp age groups/ not exp adult/ | 1851515 |
| 32 | 30 not 31 | 743 |
| 33 | (english or german).lg. | 25886158 |
| 34 | 32 and 33 | 726 |
| 35 | limit 34 to yr="2007 -Current" | 514 |
| 36 | remove duplicates from 35 | 469 |
